# Book Reviews

**Published:** 1801-07

**Authors:** 


### CRITICAL RETROSPECT

OF

### MEDICAL AND PHYSICAL LITERATURE.

---

*Henrici Aug. Wrisbergii Commentationum Medici, Physiologici, Anatomici et Obstetricii Argumenti, Societ. Reg. Scient. Gotting. Oblatarum et Editarum*, Vol. I. cum Iconibus, 1800, pp. 572, 8vo. Gottingen, Dieterich, price 3 rixd. 12 gr. or about 12s.

THE excellence and intereſt of *Wrisberg's* writings are acknowledged by every perſon, and a new edition of them, in which they are all collected from the different volumes of the Tranſactions of the Royal Society at Gottingen, muſt therefore be very acceptable to medical men. It is however to be regretted, that in this undertaking no certain order, neither of the time of their firſt appearing, nor according to the matter they treat of, has been attended to; but it appears rather from this want of order, that Mr. Wriſberg has too much left this edition to the publiſher, particularly, as we likewiſe obſerve no alterations and improvements in ſeveral papers that would have admitted of it in the preſent times. The firſt volume contains, beſides the laſt treatiſe, which has not been printed before, *twenty* ſmall papers, the merit and intereſt of which are too generally known for us to make any remarks on them. They are, 1. *De membrana fetus pupillari.* 2. *De vita fetuum dijudicanda.* 3. *De secundinarum humanarum varietate.* 4. *Comment de variolis, quibus interne corporis humani partes contaminari dicuntur, observationibus anatomicis superstructa;* to which treatiſe three new intereſting engravings are added, repreſenting pock-like eminences in the internal ſurface of the trachea, of the lungs, of the pericardium, and of the pleura. 5. *Observationes et experimenta ad pulmonum docimasiam confirmandam instituta.* 6. *Observatio anatomica de quinto pare nervorum encephali et de nervis, qui ex eadem duram matrem ingredi false dicuntur.* 7. *Observationes anatomicæ de vena azyga duplici aliisque hujus venæ varietatibus.* 8. *Dissert. de præternaturali et raro intestini recti cum vesica urinaria coalitu, et inde pendente ani defectu, Observ. anatomica superstructa.* 9. *Observat. anatomica de testiculorum ex abdomine in scrotum descensu ad illustrandam in chirurgia de hernis congenitis utriusque sexus doctrinam.* 10. *Observ. anatom. de ganglio plexuque semilunari in abdomine et nervisillum formantibus.* 11. *Experimentor. et observation. anat. de utero gravido, tubis, ovariis et corpore luteo quorundam animalium cum iisdem partibus in homine collatis, Sect. I.* 12. *Observatio anatomico obstetricia de structura ovi et secundinarum humanarum inpartu maturo et perfecto collectæ.* 13. *Comment. de membranarum ac involucrorum corp humani, partim dubiis, partim veris.*

*ris.* 14. *Observat. anatomica de nervis arterias venasque comitantibus.* 15. *Observatio anatomico medic. de nervis pharyngis.* 16. *Comment. de uteri mox post partum naturalem resectione peracta non lethali, observatione rarissima illustrata, cum brevi fundamentorum lethalitatis sciagraphia.* 17. *Observat. anat. med. de systemate vasorum absorbente, morbos vicissim excitante et sanante.* 18. *Comment. de singulari genitalium deformitate in puero hermaphroditum mentiente.* 19. *Observat. anat. neurolog. de nervis viscerum abdominalium.* 20. *De nervis systematis coeliaci, Sect. I.* 21. *De nervis gastricis, quae est observationum de ganglio plexuque semilunari continuatio, 1ma.* The laſt of theſe treatiſes contains a very intereſting contribution to the hiſtory of the inteſtinal nerves, in which Prof. Wriſberg gives an account of the nerves that proceed from the ſemilunare ganglion. He premiſes ſome pathological and anatomical remarks on the different organs of the abdomen, which he divides into four ſyſtems, *systema coeliacum, meseraicum, renale,* and *genitale,* of which the two former and the two latter have a great relation to each other. The firſt, however, differs from the ſyſtema meſeraicum, by all the organs that belong to it receiving blood from one artery only, whereas thoſe of the latter have it by two arteries. The organs of the firſt ſyſtem are ſituated in the upper region of the abdomen, juſt under the diaphragma, by the motions of which they are greatly influenced, whereas thoſe of the ſecond lie more diſtant from it in the lower region. The former ſecretes the humours for digeſtion, and prepares the nutritive matter, which is abſorbed by the other ſyſtem. When any organ of the ſyſtema coeliacum ſuffers, all the other organs are likely to be affected; it receives likewiſe a great number of nerves from the par vagum. Prof. Wriſberg divides the nerves of the ſyſtema coeliacum into the *plexus gastricos,* the largeſt of all, *plexus omentales, hepaticos, pancreatico-lienales,* and *pancreatico-duodenales.* The two firſt of theſe plexus are moſt excellently deſcribed here; but as we cannot communicate this deſcription to our readers, we beg leave to point out ſome of the reſults, as being extremely intereſting. The left part of the ſtomach has a greater conſent with the nervus vagus and the organs that receive branches from it, viz. the lungs, the pharynx, the tongue, and the trachea, than the right; which, however, has a nearer relation to the nervus intercoſtalis, the liver, duodenum, pancreas, &c. All parts of the ſtomach are not equally provided with nerves, the cardia has the moſt of them, on which account it enjoys the greateſt ſenſibility. Theſe nerves do almoſt all end in the pars villoſa, which therefore is moſtly affected by a poiſonous and other deleterious ingeſta. The inner ſides of the ſtomach poſſeſs likewiſe a great quantity of nerves, and the latus exterius more than the latus poſterius. The little curvature has a great number of nerves, whereas the curvatura magna has but few. The omentum is provided with ſmall but numerous nerves.

---

Journal

*Journal fur Medizin, &c.* i. e. Journal for Medicine, Surgery, and Midwifery, particularly with refpect to Aitiology and Semiotic. By a Society of Phyficians. Edited by Dr. J. F. S. Posewitz, Profeffor in Giefsen, No. 2. 1800. 8vo.

The firft number of the above Journal appeared in 1799. This fecond number contains, (befides an *Introduction*, in which the author fpeaks of the ufe and neceffity of enlarging and improving the aitiology of difeafes by accurate obfervations and by the teft of experience) 1. *Obfervation on a peculiar exanthema, by Dr. Schmidt.* A child of five weeks was affected by a puftulous eruption, which returned periodically every fortnight, and difappeared without leaving any rednefs, whereby a flow fever came on, lafting till the eruption appeared again. It was fuppofed to be occafioned by fome alteration in the conftitution of the mother, who had fhortly before fuffered a very fudden fright. It was cured by mercurius cinereus, within three months, after having continued for nearly eighteen months. 2. *Obfervations on the treatment of wounds, penetrating the integuments and bones of the head; by Dr. Baumer:* The author recommends the trepan in all fiffures and depreffions of the fkull as very neceffary. 3. *On difficult dentition, by Dr. Wendelstaedt;* in which the Theory of Dr. Wichmann is refuted. 4. *On a delirium, by the Editor,* which came on in a patient who had fuffered ten years by epileptic fits. 5. Hiftory of a murder, committed by knocking the perfon on the head, without any external injury being perceptible. 6. *On the fymptoms of fevere injuries of the head, by Dr. Muller.* 7. *On difficult dentition, as far it may give rife to infantile difeafes, by the Editor.* 8. *Reviews.*

---

*Archiv fur Zoologie and Zootomie;* i. e. Archives for Zoology and Zootomy; edited by C. R. W. Wiedemann, Profeffor at the Anatomical Chirurgical College in Brunfwick, &c. Vol. I. No. 1. with engravings, pp. 208, 8vo. Berlin, for Vofs, price 1 rixdollar, or about 3s. 6d.

The combination of Zoology and Zootomy, as the author juftly remarks, may prove not only very ufeful and interefting for fyftematic inquiries, but great advantages may hence arife for phyfiological refearches, and it is on this account that the prefent publication deferves to be recommended. The author intends to lay down every thing relating to Zoology, in the wideft fenfe of the word; to infert original papers, as well as to give faithful extracts, and to collect any hint that is otherwife loft to the public. By which, indeed, he is likely to render the ftudy of thofe branches of natural fcience more convenient and eafy, and at the fame time lefs expenfive. The firft number of this Journal contains the following heads: 1. On the ftudy of comparative anatomy, by the editor. 2. On the anatomy of animals, by H. Winkelmann. 3. Effay of a comparative defcription of fkulls, from all orders of quadrupeds, by the Editor. 4. Defcription of the fkeleton of an armadilla. 5. Description of the skeleton of a sloth. 6. On the organs of digestion, with some remarks on rumination. 7. Account of Zoological publications and miscellaneous intelligence. The plates are extremely well executed; the two first represent several skulls of Bradypoda, in different views; The third gives a representation of that curious animal from New South Wales, Platypus anatinus, or ornithorhynchus paradoxus.

---

### *Mr. Charles Bell's System of Dissections.*

( Continued from Vol. V. pp. 579—581. )

" In examining the body, it will be observed how the stomach and spleen may be wounded by a thrust apparently into the thorax; or how the lungs and stomach, or lungs and liver, may be thrust through at once. It may also be observed how the stomach and liver lie before the diaphragm, where it goes obliquely down upon the back part of the abdomen; and how they lie contiguous to one another. The effect of hydrothorax, in pushing down the diaphragm, and depressing these parts—and the effect also of enlargement of the liver, or distention of the stomach upon the breathing, must be obvious. It will also be seen how a hernia of the stomach into the thorax may happen by a rupture of the diaphragm.

" The stomach is commonly retired behind the colon, and under the ribs. Yet when slightly distended, it comes further down in the belly, and assumes the place of the arch of the colon. Therefore finding a patient with an open ulcer immediately under the scorbiculus cordis, discharging the contents of the intestines (an instance of which I have lately seen), it may be questioned whether it be an opening from the stomach or from the colon. This may perhaps be determined by observing the matter discharged, whether it be food partly digested, as from the stomach; or fæces, as from the colon, after having gone through the whole length of the canal.

" Ulceration of the inner coats and cancerous tumours of the stomach are frequent. Upon the inside of the stomach, intestines and bladder, when violently inflamed by any irritation, there is often a general redness with bloody slime, or there are small blots of florid extravasation. The few cases of cancerous tumours of the stomach which I have seen, accord with the common observation, that the upper orifice of the stomach is more liable to disease, (owing to its more glandular structure) and more exposed to injury from substances swallowed. I have seen the whole upper half of the stomach, with its walls so much diseased, that they were not less than one inch in thickness, with their inner surface soft and cancerous. This disease was attended, during the patient's life, with a continual gnawing pain, with soreness even upon taking the softest food, and with extreme pain upon swallowing fwallowing any thing, in the flighteft degree ftimulating. In difeafes of this kind, and where there are tumours projecting into the ftomach, there is frequent vomiting of black chocolate-coloured blood, which is often attended with fainting.

" Contractions of the œfophagus, and upper orifice of the ftomach, are frequent without any organic difeafe. When there is difeafe of the œfophagus, the ftomach is found contracted in breadth, and fcarcely to be diftinguifhed from the inteftines, (Haller); but it is faid, that when the difeafe is lower down, the ftomach is found to be diftended. I do not know whether this laft obfervation holds good. The ftomach, however, is often diftended fo as to fill almoft the whole belly, without any other extraordinary appearance. And in thefe cafes the fmall inteftines being compreffed, obftinate coftivenefs is induced.

" That the coats of the ftomach are affected by its own juices, is now univerfally believed. But, as far as I have obferved, erofion is not in fuch cafes to be expected. Nor are the coats thinner, but they are thickened, gelatinous, yielding, and tender to the fingers; a circumftance that has often troubled me in injecting the veffels of the ftomach. That this is produced by the fecreted fluid of the ftomach, unaffimilated with the food, we are affured from the inteftines not being thus affected, though in the fame circumftances with regard to putrefaction. Although it be a common and well founded obfervation, that the ftomach is extremely delicate, and although the inftances of fudden death occafioned by blows upon it are very frequent, yet there are fome cafes that would feem to form exceptions to this. A young man received a kick in the belly from a horfe. It occafioned long a conftant pain in the fore part of the belly, weaknefs and indigeftion; thefe were fucceeded by a tedious hectic fever, and at laft proved fatal. Upon diffection, the omentum was found folded up and contracted round the ftomach, forming a folid mafs of about an inch and a half in thicknefs, and connecting the ftomach and inteftines and liver by its adhefions. The ftomach itfelf was turned to a bloody grumous cancer. The outlines of this cafe will point out the difficulties which will fometimes occur in unravelling thefe parts when difeafed.

" Obferve the fituation of the liver towards the right fide; how far it comes down into the right hypochondrium; and how dangerous and improper it confequently is to tap on this fide, the more efpecially as the liver is often enlarged in dropfy. Obferve, again, the clofe connection of the liver with the diaphragm, and how abfceffes, originally formed in the liver, may, by the fpreading of the inflammation, and by the adhefions with the diaphragm, communicate the fuppuration to the lungs, fo that the matter from the liver may be coughed up from the breaft; or how hydatids originally formed in the liver may, by the fame communication, be coughed up from the lungs; or how matter in the liver may, by its natural tendency to the furface, propagate the inflammation to the abdominal mufcles, and, by forming adhefions with them, be difcharged outwardly. In this laft cafe the adhefions, always preceding the formation and progrefs of matter outwardly, the attachment of the liver and integuments is clofe and intimate, and the abfcefs points regularly, fo that the operation is very eafy. Abfcefs of the liver, befides being attended with a peculiar painful feeling in the right hypochondrium, is accompanied with a fharp pain of the fhoulder and clavicle of the fame fide ; yet it fometimes happens, that the liver is fo little fenfible, that upon diffection, there are found great abfceffes where the patient, during life, had no complaint. There are, in the writers upon the difeafes of hot climates, fome ftrange examples of the extenfive communications of thefe abfceffes.

" After having obferved the intimate connection of the liver, duodenum, and ftomach, it is eafy to conceive a cafe which not unfrequently happens, viz. a difcharge of matter into the ftomach and inteftines, and even a difcharge of the food by the external wound, after an operation for abfcefs of the liver; for it has happened, that the abfcefs of the liver has formed connections with the ftomach on the one hand, and, on the other, opened outwardly upon the fide of the belly. It will alfo be feen how hydatids, getting entangled with the inteftines, may be difcharged by ftool; and how tumours of the liver, pancreas, and fpleen, muft opprefs the ftomach.

" With regard to the operation for the collections of matter in the liver, unfortunate miftakes have been made. There is a cafe mentioned by Haller, of what he calls a fpurious aneurifm, in which, upon the tenth rib below the fcapula, and in the mufcular flefh of the back, there feemed to be the pointing of an abfcefs, which yielded to the fingers ; the patient having at the fame time a flow fever, and a jaundiced complexion. They had no doubt of its being an abfcefs of the liver ; but the patient died of the violent hæmorrhagy the night following the operation. There is another cafe which brings home to us ftill more forcibly the importance of an accurate knowledge of thefe parts, and of a lively conception of the effects of difeafe upon them. In l'Hofpital de la Charité in Paris, the operation for empyema was performed, but no matter flowed from the incifion. They had been deceived chiefly by the circumftance of matter being fpit up from the lungs. Upon diffection, they found that the matter had been originally formed in the liver, and from it had been communicated to the lungs; but that this communication, having been formed deep in thefe vifcera, no matter could flow from the incifion. In Ruyfch there is another cafe of a country furgeon cutting into the liver, when operating for paracentefis of the thorax; and the cafe fhows, at the fame time, the poffibility of miftaking enlargement of the liver with hydatids for hydrothorax. See in Sandifort (*Obser. Anat. Path.* Lib. III. Cap. V. p. 83. Not. e.) a curious cafe of abfcefs; and indeed inftances of fuch cafes are very numerous. It has been found, too, that fuch is the fympathy between the ftomach and liver, that the dreffings after the operation operation for abfcefs being too much ftuffed into the wound, have occafioned violent bilious vomiting, which was removed upon withdrawing them.

"In diffection, there is frequent occafion to remark the foftnefs of the liver when difeafed; and it is neceffary to obferve its colour when not difeafed, fo as to be able to judge in any other inftance how far its colour is natural. Often, in difeafe, it is of a lighter colour, or fpotted and marbled, or its thin edges are found tinged with blood as if inflamed, or perhaps they are found livid, which may fometimes be produced by the pofition of the body after death, and the gravitation of the blood, as happens in the lungs. At any rate, there is feldom active inflammation of the liver. It is often fchirrous and enlarged, and then afcites is frequently combined. Its fchirrous ftate, when far advanced, is palpable enough; it feels knobby and irregular on the furface, and, when cut, the tubercles are generally of a light brown colour. See varieties of thefe in Baillie's Morbid Anatomy. The liver, the kidney, the fpleen, and the uterus, that is, all the folid vifcera, feem peculiarly the feat of hydatids, but particularly the liver; and from the burfting of the parent facs, fituated in thefe parts, the fmaller veficles get entangled with the membranous vifcera.

"The laft circumftance that feems worthy of notice in this part of the belly, is the obliquity of the diaphragm, and the manner in which the parts lie upon it. If there be a tumour formed in any of thofe parts that are protruded by the action of the diaphragm, fuppofe an aneurifm of any of the veffels, however the furface of the belly may be moved, the pulfation of this tumour will be continual upon the hand; but if the tumour be fituated upon any of the veffels whofe attachment to the fpine hinders them from being difplaced by the motion of the diaphragm, then the action of the diaphragm, and confequent protrufion of the vifcera and integuments of the belly, will give the feeling as if its pulfation were fubfiding while the tumour retires from the hand. This circumftance, fimple as it is, is the more apt to be overlooked, as a patient, when a phyfician feels his belly, does not breathe regularly, but ftrains himfelf, and breathes at intervals."

---

*A Practical Essay on the Art of recovering Suspended Animation; together with a Review of the most proper and effectual Means to be adopted in Cases of imminent Danger.* Tranflated from the German of CHRISTIAN AUGUSTUS STRUVE, M.D. 12mo. pp. 200. London, 1801, Murray and Highley.

OUR readers are already acquainted with the merit and abilities of Dr. Struve,* and the prefent work does in no degree derogate

 from

---

* See Journal, Vol. V. pp. 293—377.

from them. This comprehensive practical work deserves to be generally distributed at the public expence: No medical practitioner, magistrate, public inn, or farm house, should be without it.

It is divided into three sections; in the first of which Dr. S. gives the history of *humane* institutions, the signs of life, the signs of death, the causes or accidents which produce apparent vital extinction, such as suffocation by water, by vapours, by a cord, &c. cold, lightening, violence, &c. In this part also the author examines the general principles of the resuscitative process.

In the second section we find an account of the resuscitative apparatus, and the practical rules, from which we present our readers with the following extracts.

" To those who wish to become more intimately acquainted with the most perfect apparatus for this purpose, I recommend the following excellent publication: " *Geschichte und jetzige Einrichtung der Hamburgischen Rettungsanstalten für im Wasser verunglückte*," namely, " The History and present State of the Resuscitative Institution of Hamburgh:" by J. A. Gunther, with plates, Hamb. 1794.

" In order to render this work more perfect, I have also mentioned Gorcy's bellows, though I am convinced, that they are almost unnecessary: for the principal attention of the practitioner should be directed to a speedy excitement of irritability.

" I. *Resuscitative Apparatus.*

" A. *General apparatus.*—1. *Instruments:* A lancet, bistoury, a syringe to cleanse the throat of mucus; blankets.—2. Sticking plaster.—3. Soft brushes for friction.—4. A quantity of brandy or wine.—5. A bottle of oil.—6. Vinegar.—7. Culinary salt.—8. Warm water for bathing.—9. Cold water.—10. A deep tub for bathing the feet.—11. An oblong vessel for bathing the whole body.—12. A quantity of aromatic plants.—13. Ginger, mustard, vesicatories.

" B. *Particular instruments.* 1. Hunter's thermometer.—2. Three or four small tubes, such as Coleman has recommended.—3. Gorcy's bellows.—4. A portable electric machine. 5. Dr. Richter's instrument for opening the wind-pipe.

" *A particular chest*, containing the most necessary instruments, and a sufficient quantity of medicines for internal and external use, ought to be in the possession of every medical man.

" II. *Practical Rules for the Treatment of Persons apparently dead, or endangered.*

" 1. Crowds should be guarded against, as five or six persons are sufficient to perform the process, and a few others may procure the necessary instruments.

" 2. The person endangered should not be brought into a warm or crowded room.

" 3. A free circulation of air must be obtained, by opening the windows of the room where the process is conducted.

" 4. A

" 4. A hafty application of refufcitatives is dangerous, and tends to extinguifh the vital principle.

" 5. All refufcitative remedies, when too ftrong, and abundantly adminiftered, are prejudicial.

6. Proper means of exciting fufceptibility ought to be applied from the commencement, and continued throughout the whole procefs of refufcitation.

" 7. Stimulants fhould be adminiftered only in proportion to the perceptible degree of fufceptibility.

" 8. All refufcitatives are to be gradually applied, according to the temperature of the body.

" 9. A fingle remedy fhould never be confidered as fufficient to reftore animation, but a proper and fucceffive application of feveral fhould be purfued.

" 10. Before the patient has recovered the power of deglutition, neither medicine nor nutriment ought to be adminiftered. Emetics, in particular, might prove fatal at this period.

" 11. Clyfters of tobacco, emetics, and fternutatories, ought to be ufed with great caution, or not at all; they are particularly dangerous at the commencement of the procefs.

" 12. All powerful odours are prejudicial, efpecially during the firft indications of life.

" 13. The procefs fhould be continued from *four* to *fix* hours; nor fhould the recovery of the patient be defpaired of, till the moft unequivocal figns of death have become evident.

" III. *Particular Directions.*

" *Treatment at the commencement.*—1. Violent concuffions are prejudicial, though a moderate rolling motion has fometimes been efficacious.—2. Strong friction is alfo injurious at the commencement of the procefs.—3. Particular care is required to wrap the body in warm blankets or cloths, and warm it moderately, in cafes where a higher temperature is falutary.

" *Continuation of the Treatment.*

" 1. The procefs of warming, and the means of exciting fufceptibility, muft not be too foon difcontinued; nor fhould the perfon apparently dead, be uncovered, but when indifpenfably neceffary.

" 2. Stronger friction and ftimulants are generally indicated;

" a. By figns of fufceptibility; fuch as returning warmth; throbbing of the heart, and fpafmodic fhivering of the limbs.

" b. The method of exciting fufceptibility of irritation having been continued for fome time, it may be fuppofed actually to exift, and fuch ftimulants are to be adminiftered, as may tend to promote its developement.

" 4. Gentle ftimulants ought to be firft adminiftered, and thefe of a ftronger nature gradually and fucceffively applied.

" *Treatment*

"*Treatment of the Patient on the first Symptoms of returning Life.*

"1. A precipitate method to enforce refufcitation ought to be guarded againft, as well as the application of violent ftimulants, fuch as ftrong odours, &c.

"2. The before-mentioned method, particularly that of gentle friction, is to be moderately continued.

"3. The gradual developement of the vital power fhould be carefully attended to, as the nature and degree of ftimulants muft be determined in confequence.

"4. A careful continuance of the application of warmth to the patient will be moft efficacious at this period.

"IV. *General Treatment of Persons in Danger.*

"In order to enable the reader to comprehend, at one view, all refufcitatives, and the rules for their application, with refpect to vital power, I fhall give a fhort defcription of the treatment of perfons endangered by fatal accidents.

"*First Treatment.*

"Diligent fearch for the body, and its removal to the open air; undreffing, &c.

"The fubject fhould be placed in a horizontal pofition; the head fomewhat elevated, and turned to the left fide; the beft place is a table in the middle of a room, fo that the affiftants may have eafy accefs.

"The perfon apparently dead, ought alfo to be removed in the above mentioned pofture, and the mouth and nofe cleanfed of froth or filth without delay, to facilitate the communication of air to the lungs. This may be effected by the finger, a feather dipped in oil, or by a fyringe. The body is, at the fame time, to be kept covered with warm blankets."

Thefe general rules are then explained at length, and the treatment of each cafe particularly laid down.

The Third Section contains the means of faving perfons in extreme danger from accidents, poifons, apoplexy, &c. with an account of the different kinds of poifons and their antidotes, the means of detecting fugar of lead in wine, &c.; for all which we muft refer to the Work itfelf.

---

GOTTFRIED CHRISTIAN REICH, *Med. et Chirurg. Doct. et Professor, Member of many learned Societies,* ON FEVER, *and its Treatment in general. Published by command of the King of Prussia, by the Higher College of Medicine and Health of Berlin,* 1800. *Translated from the German, by* CHARLES HENRY PARRY, *Ordinary Member of the Physical Society of Goettingen. To which are added, a Preface, by the Translator; and an Appendix, by* CALEB HILLIER PARRY, M. D. F. R. S. *Member of the*

*the Royal College of Physicians of London; and one of the Physicians of the General Hospital of Bath.* 8vo. pp. 102. London, 1801. Cadell and Davies.

FOR an account of the original of this work, fee Journal, Vol. iv. p. 556, and Vol. v. p. 178.

From the Appendix by Dr. Parry, we make the following extract.

" As I have reafon to fuppofe the preceding pages to be an accurate tranflation of a work, which has been fo much fought for in Germany, that the Tranflator has not been himfelf able to purchafe a copy, I fhall not attempt to reconcile any apparent contradictions in the theory, or make on it any further remark, than that the operations on the human œconomy, attributed to oxygen and azot, appear to be totally oppofite to thofe which are affumed by the Englifh chemifts. Which of thefe opinions is juft, future experience muft decide. The moft important point is the matter of fact; whether the acids are really capable of producing, in all or any febrile difeafes, thofe beneficial effects which are promifed from their ufe. If this fhall eventually prove to be the cafe, and it fhall appear that the practice has been derived by fair deduction from chemical principles, we may congratulate ourfelves on having at length extracted fome gold from the ordure of medical chemiftry.

In the work itfelf, there is a great air of earneftnefs and ingenuoufnefs. The author candidly acknowledges, that the ufe of acids in fevers had been eftablifhed by old and long experience; but he obferves that they were employed in very fmall quantities, and only as auxilliaries; far from being confidered as of primary and effential confequence in the cure. To this it may be added, that the acids which have been chiefly exhibited, have been either the vitriolic, or, more efpecially, thofe of the vegetable kind. The ufe of the muriatic acid has been more rare, though not wholly unknown. As long ago as the year 1773, that acid was highly recommended by our countryman, Sir William Fordyce, to be given internally in thofe fevers which were called putrid or malignant; and to be applied externally in the form of liniment or gargle to the floughs in the throat, which often accompany fuch fevers. His liniment confifted of twenty drops of the concentrated acid to an ounce of honey of rofes; and his febrifugum antifcepticum contained five drops of the acid mixed with two ounces of a ftrong decoction of Peruvian bark. In a fubfequent publication, which appeared in the year 1790, in form of a letter to Sir John Sinclair, Bart. " concerning the virtues of the muriatic acid," he exprefsly recommends that medicine as the beft remedy in " all our putrid difeafes of the worft kinds;" " in petechial, camp, and jail diftempers, as well as the malignant fore throat, fo frequently fatal in this country; and afterwards in the fmall-pox and plague. In this letter, the author acknowledges himfelf indebted for the muriatic gargle to Sir William Duncan; and quotes a pamphlet on the external and internal ufe of this acid, publifhed in the year 1664,

1664, by Conſtantine Rhodocanacides, a Greek empiric, who calls himſelf chemiſt to King Charles the Second, and who derives his theoretical preſumption of its extraordinary power from the univerſality and approved value of common ſalt. He recommends the acid, mixed with food and drink to the amount, if neceſſary, of one hundred drops in the twenty-four hours, as a preventive and remedy for the plague, and as a general antiſceptic; and publiſhes, at the ſame time, ſeveral "certificates of the great benefit that had been received from it." To this ſource Sir William Fordyce confeſſes himſelf indebted for the firſt hint, reſpecting the internal uſe of the muriatic acid in putrid diſeaſes; and he enumerates ſome caſes of that kind, in which he attributes the cure chiefly to that acid.

"In the form of glyſters, I do not know that the muriatic acid has been preſcribed in England by any other perſon than myſelf. In this manner I, ſeventeen years ago, employed it, mixed with nitrate of ſoda, apparently with very good effects, in ſeveral caſes of the typhus, in the Pauper Charity in this city. But as the adminiſtration of the muriatic acid in theſe inſtances aroſe merely from a chemical miſtake, I never purſued that practice; nor have I learned that the publication of Sir William Fordyce has induced one ſingle phyſician in this country to truſt to the internal uſe of the muriatic acid for the cure of any Febrile diſeaſe."

---

*The Medical and Chirurgical Pharmacopœia, for the Use of Hospitals, Dispensaries, &c.* By RICHARD REECE, Chepſtow. 8vo. p. 88. Briſtol, 1800. London, Weſt and Hughes.

THAT our readers may be enabled to judge of the Author's views in this publication, we give an extract from his Preface.

"It was a wiſe and beneficent ſpirit that directed ſuch amiable inſtincts to the patronage of Inſtitutions which might, at all ſeaſons, afford to diſeaſe a conſolatory refuge in time of need; and we may with ſome triumph exult in the conviction, that the medical profeſſion of our own country, know their nature better than to uphold ſuch eſtabliſhments from any principle of mercenary gratification. In moſt other States, I believe this eulogium cannot ſo truly and judiciouſly apply to that rank of its members. The opinion, however, is by no means ſingular, that an opening may ſtill be left in the improvement of their economy. In the humble hope, therefore, of giving riſe to a plan which may retrench in Infirmaries the ſcale of general expenditure, without, at the ſame time, with-holding any ſuccour for the relief of individual malady, the following little Treatiſe has been drawn up to meet the public eye. It is requeſted that the medical profeſſion will not conſider the intention of the author as originating from any deſire of *preſcribing* to their judgment, or cenſuring the prevailing ſyſtem, but only as a hint, if the alteration ſhould be deemed expedient or neceſſary, on which gentlemen of more enlarged practice and ſuperior talent may improve. It has been at times thought convenient to depart from from fome of the ufual names for the fake of a more diftinctive perfpicuity. In order to fwell as little as poffible the contents and price of the book, brevity has been uniformly adhered to wherever it could be done with effect, and the few ftray notes which run through the volume, are only fuch as appear to the author indifpenfibly neceffary."

We think the Author has deferved well of the Profeffion by this Effay, and we have no doubt that his benevolent intentions will be confiderably promoted by its publication.

---

*Considerations regarding Pulmonary Consumption.* By T. Sutton, M. D. 8vo. pp. 84. London, 1799, Robinfons.

Although the greateft part of this work was printed in December, 1799, as we are informed in the appendix, it was publifhed only a few weeks ago. The leading feature of this performance is the novelty of the *caufe* of Pulmonary Confumption, which the author ftates in thefe terms.

"The firft fymptoms of difeafe in the cafe related, were in the bowels, and by degrees the diforder became a confirmed phthifis pulmonalis. Hence, through its progrefs, feeing every pulmonary fymptom fo mild, I was often led to fufpect *the emaciation and debility to be induced by some disease of the abdominal viscera*; which, however, I could not account for in any other way, except by fuppofing the mefenteric glands to be obftructed, as the fymptoms led to no fufpicions of any other caufe or caufes that could be confidered as adequate to produce fuch effects.

"During the progrefs of the cafe firft related, I infpected the bodies of two foldiers after death, at the Southampton Military Hofpital, who had been firft affected with dyfentery, and afterwards with pulmonary confumption; in both which there were indurations in the mefenteric glands, with tubercles in the lungs. I have, fince then, feen feveral cafes of confumption, where affections of the bowels preceded the pulmonic fymptoms. It is a very common thing for patients, in protracted dyfenteries, to have pulmonic affections before death: Of fuch cafes I have feen many in the general military hofpitals; and it frequently happens that difeafes of the abdominal vifcera are in their latter ftages accompanied by pulmonary confumption. By writers on this difeafe, the tabes mefenterica is mentioned as fometimes accompanying it.

"From a confideration of thefe circumftances, I was more ftrongly impelled to believe, that an obftruction of the mefenteric glands was the caufe of pulmonary confumption, or at leaft was the principal caufe in occafioning the deaths of the patients, as no other adequate caufe of the fatality of the difeafe, and of fome of its principal phænomena, appeared to me to have been affigned. For, from what has been faid of the effects of hectic fever, it was evident to me that it could not be of fo pernicious a nature; and the mild pulmonary fymptoms in many cafes can by no means be taken into account as the caufes of death.

" I have before ftated the ultimate caufe of the death of patients in confumption to be a want of proper ftimulus in the blood to carry on the circulation, and that it may be accelerated by a difeafe in the lungs.

" Hence it appears to me, that phthifis pulmonalis is caufed by a difeafe in the mefenteric glands, and that the tubercles in the lungs, and fome other of its fymptoms, are excited by fympathy.

" It may be here alleged, that a fympathetic confumption arifing from difeafes in the abdominal vifcera has long been admitted, and that it is probable that fuch diforder may be communicated in the manner juft ftated; but it will be contended, that the fource of phthifis is very often in the lungs themfelves; which is evidenced by the affection being excited by extraneous fubftances inhaled into that vifcus; as happens to ftone-cutters, fciffor-grinders, and others employed in fimilar occupations. Such opinions I know to have received very confiderabe credit amongft medical men; and thofe appear to be the faftneffes in which they may hold out, that the affection of the lungs, called pulmonary confumption, is often, or (as will be generally afferted) moftly, an idiopathic difeafe.

" We are not fufficiently acquainted with the laws of the nervous fyftem, to be able to give any idea refpecting their mode of action, in occafioning difeafes of fympathy, though there is no principle more generally admitted than this. There are two affections of parts diftant from the lungs, which muft be accounted for in this way in pulmonary confumption, according to the prefent idea that the lungs are primarily affected. The one is the affection of the mefenteric glands, and the other of the glands of the neck: for in no other way can thefe affections be accounted for, except by the influence of this principle. On this account it will be lefs furprifing, if I introduce it, to connect the difeafe of the mefenteric glands as a primary difeafe with that of the lungs. For, as in the former cafes, it has been long admitted, fo, in the other, fhould it appear probable, that the mefenteric glands may be firft affected, there can be no difficulty in admitting, that they may by fympathy be capable of occafioning tubercles and other affections of the lungs. Befides, it has long been allowed that pulmonary confumption is fometimes a fecondary difeafe.

How much foever writers may differ in their theories and caufe of difeafes, we generally find them agree in the cure. Dr. S. however, has but litttle faith in Digitalis, and follows Dr. Reid in placing his principal reliance on emetics. " For an increafed action may be produced by exciting an increafed motion in the contiguous parts, which may be effected by the ufe of emetics and purgatives, which promote a greater motion in the inteftinal canal, and from their contiguity, in all probability, communicate fome of it to the mefenteric glands. And as I conceive that emetics not only excite greater motions in the inteftines, where the glands are moft affected, but produce a determination to the external parts, and appear to me to caufe in no wife fo great a debility, I fhould recommend the general ufe of them in preference to purgatives."

